# Quasi‐Diffusion Imaging: Application to Ultra‐High *b*‐Value and Time‐Dependent Diffusion Images of Brain Tissue

**DOI:** 10.1002/nbm.70011

**Published:** 2025-02-28

**Authors:** Thomas R. Barrick, Carson Ingo, Matt G. Hall, Franklyn A. Howe

**Affiliations:** ^1^ Neurological Disorders and Imaging Section, Neuroscience and Cell Biology Research Institute, School of Health and Medical Sciences City St George's, University of London London UK; ^2^ Department of Neurology Northwestern University Chicago Illinois USA; ^3^ Department of Physical Therapy and Human Movement Sciences Northwestern University Chicago Illinois USA; ^4^ Medical, Marine, and Nuclear Department National Physical Laboratory Teddington UK

**Keywords:** brain, diffusion magnetic resonance imaging, diffusion signal representation, non‐Gaussian diffusion imaging, quasi‐diffusion imaging

## Abstract

We demonstrate that quasi‐diffusion imaging (QDI) is a signal representation that extends towards the negative power law regime. We evaluate QDI for in vivo human and ex vivo fixed rat brain tissue across b‐value ranges from 0 to 25,000 s mm^−2^, determine whether accurate parameter estimates can be acquired from clinically feasible scan times and investigate their diffusion time‐dependence. Several mathematical properties of the QDI representation are presented. QDI describes diffusion magnetic resonance imaging (dMRI) signal attenuation by two fitting parameters within a Mittag–Leffler function (MLF). We present its asymptotic properties at low and high b‐values and define the inflection point (IP) above which the signal tends to a negative power law. To show that QDI provides an accurate representation of dMRI signal, we apply it to two human brain datasets (Dataset 1: 0≤b≤15,000 s mm^−2^; Dataset 2: 0≤b≤17,800 s mm^−2^) and an ex vivo fixed rat brain (Dataset 3: 0≤b≤25,000 s mm^−2^, diffusion times 17.5≤∆≤200 ms). A clinically feasible 4 b‐value subset of Dataset 1 (0≤b≤15,000 s mm^−2^) is also analysed (acquisition time 6 min and 16 s). QDI showed excellent fits to observed signal attenuation, identified signal IPs and provided an apparent negative power law. Stable parameter estimates were identified upon increasing the maximum b‐value of the fitting range to near and above signal IPs, suggesting QDI is a valid signal representation within in vivo and ex vivo brain tissue across large b‐value ranges with multiple diffusion times. QDI parameters were accurately estimated from clinically feasible shorter data acquisition, and time‐dependence was observed with parameters approaching a Gaussian tortuosity limit with increasing diffusion time. In conclusion, QDI provides a parsimonious representation of dMRI signal attenuation in brain tissue that is sensitive to tissue microstructural heterogeneity and cell membrane permeability.

## Graphical abstract

Quasi‐Diffusion Imaging (QDI) signal transitions from stretched exponential to negative power law behaviour at ultra‐high *b*‐values via an inflection point (data and fits shown for representative (a) grey and (b) white matter voxels). QDI is parameterised by the (c) diffusion coefficient, *D_1,2_* (in mm^2^s^−1^) and (d) apparent exponent, *α*, allowing computation of the (e) *b*‐value of signal inflection. Time dependence of (f) *D_1,2_* and (g) *α* in fixed rat brain is demonstrated for Δ = 17.5, 50, 100, and 200 ms.

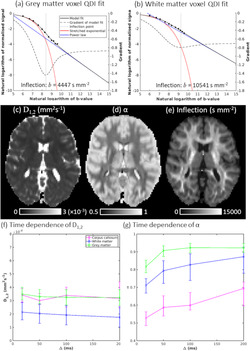

**Abbreviations List**
δ: diffusion gradient duration
Δ: diffusion gradient separation
ADC: apparent diffusion coefficient
CC: corpus callosum
CSF: cerebrospinal fluid
CTRW: continuous time random walk
DBSI: diffusion basis spectrum imaging
DKI: diffusional kurtosis imaging
dMRI: diffusion magnetic resonance imaging
GM: grey matter
IP: inflection point
ILT: inverse Laplace transform
MLF: Mittag–Leffler function
NEXI: neurite exchange imaging
NODDI: neurite orientation distribution and density imaging
pdf: probability density function
PGSTE: pulsed gradient stimulated echo
QDI: quasi‐diffusion imaging
ROI: region of interest
SANDI: soma and neurite diffusion imaging
eSANDIX: soma and neurite diffusion imaging with exchange.
SNR: signal‐to‐noise ratio
srPGSE: single refocussed pulsed gradient spin echo
TE: echo time
TR: repetition time
VERDICT: Vascular, Extracellular, and Restricted Diffusion for Cytometry in Tumours
WM: white matter

## 1 Introduction

Ultra‐high b‐value diffusion magnetic resonance imaging (dMRI) has the potential to provide more accurate and sensitive detection of brain tissue microstructural characteristics in healthy and pathological tissue [1, 2, 3, 4]. For moderate diffusion‐sensitisations (1000≤b≤3000 s mm^−2^), dMRI signal attenuation in tissue can be approximated by the second‐order cumulant expansion, a signal representation provided by diffusional kurtosis imaging [5, 6] (DKI), or by the stretched exponential [7, 8], which depending on its form can be considered a model of space‐fractional diffusion [9] or fractional motion [10, 11]. These signal representations approximate a transition from a Gaussian to non‐Gaussian diffusion as the ensemble of diffusing water molecules explore heterogeneous tissue microstructure within the diffusion time. At high to ultra‐high b‐values (3000≤b≤25,000 s mm^−2^), a negative power law decay is observed in directionally averaged dMRI signal [3, 12, 13] that cannot, in general, be explained by a simple extension of these signal representations. Herein, we refer to the negative power law exponent as α.

The negative power law arises from an impermeable intraneurite space dominated by myelinated axons that leads to a negative power law exponent of α=1/2 as b→∞ (e.g., [14, 15, 16, 17]), which was first demonstrated by Novikov et al. [18, 19] and is related to the Debye–Porod law of diffractive behaviour in porous media [20]. Experimental studies of brain white matter (WM) tissue microstructure report α≈1/2 providing support to model predictions [3, 12, 13] and can be explained by the spherical mean of the Gaussian diffusion tensor model [21], and its higher order correction can be extended by the kurtosis term [3]. However, in grey matter (GM), α≈0.8 is reported, predominately due to a lack of myelinated axons [12]. Consequently, GM tissue compartment models have been developed that include intraneurite, intrasoma and extracellular compartments (Soma and Neurite Diffusion Imaging [22], SANDI), an intraneurite and a soma/extracellular space compartment with exchange via a Kärger model (Neurite Exchange Imaging [23], NEXI) or a SANDI compartment model with exchange [24] (eSANDIX).

By varying the diffusion time, t, of dMRI data acquisition, the properties of tissue microstructural restriction can be probed at different length scales potentially allowing classification of tissue microstructural properties based on temporal power laws [16, 18, 19, 25, 26]. The mechanisms underpinning these results are encompassed within an effective medium theory that relates cell size and diffusion length scales such that diffusion parameters approach a tortuosity limit as diffusion time increases and course‐graining dominates the signal [16, 18, 19, 25]. Time‐dependent changes in diffusion coefficient, D, and kurtosis, K, have been observed in brain tissue where $D\left(t\right)$ decays to a constant, $D_{\infty }$, and $K\left(t\right)$ tends to 0 as t→∞, representing a Gaussian tortuosity limit [24, 26, 27, 28, 29, 30, 31, 32] with tissue microstructure and cell permeability being factors in the rate of this transition.

Quasi‐diffusion imaging (QDI) has been developed [33, 34, 35] from a stochastic model of diffusion dynamics that describes an ensemble of random walkers within an image voxel as a continuum from stretched exponential signal attenuation at low b‐values to a negative power law at high b‐values. Instead of modelling a signal based on an assumed set of discrete compartments with additional factors such as permeability, the quasi‐diffusion process is represented as a continuous time random walk (CTRW) with a distribution of step lengths and waiting times, which for quasi‐diffusion are coupled and represent a Gaussian mixture of walkers [36] that gives rise to a normal effective diffusion coefficient and a mean squared displacement that linearly increases with time [33, 34]. Although CTRW models have been shown to be applicable to dynamics of animals [37], cell migration [38], diffusion in crowded environments [39] and movement of large molecular tracers in proteins [40], cytoplasm [41, 42] and brain tissue [43], these have not yet been fully justified in application to water molecules in tissue microstructure.

The quasi‐diffusion approach provides a parsimonious description of dMRI signal attenuation as a stretched Mittag–Leffler function [44] (MLF) with only two parameters [33, 34]: the quasi‐diffusion coefficient, $D_{1,2}$ (in mm^2^ s^−1^) and a fractional exponent, α. $D_{1,2}$ and α are both dependent on tissue microstructure, with $D_{1,2}$ being similar in magnitude to the conventional apparent diffusion coefficient (ADC) and α representing the ensemble‐level effects of microstructural barriers. α is also the negative power law exponent of the signal attenuation at high b‐values [34]. QDI parameters have been shown to be sensitive to age‐related tissue microstructural changes in the corpus callosum [45] and pathologies such as amyotrophic lateral sclerosis [46], small vessel disease [33] and brain tumour [33]. High‐quality tensor maps of $D_{1,2}$ and α have been obtained in clinically feasible acquisition times from only 13 diffusion‐weighted images [33] (a b = 0 s mm^−2^ and two non‐zero b‐values in six directions) for which an acquisition protocol has been optimised for a standard clinical MRI system [35] with b‐values up to b=5000 s mm^−2^.

In this study, we investigate whether QDI describes a much larger range of dMRI signal attenuation in brain tissue to include an ultra‐high b‐value range (up to b=25,000 s mm^−2^), which includes b‐values with power law behaviour of the MRI signal. We investigate how QDI provides information regarding power law behaviour whilst defining a transition towards a negative power law regime. This transition is characterised by an ‘inflection point’ (IP) in the gradient of the logarithm of the signal decay. We present theory that includes the limiting equations for quasi‐diffusion as b→0 and as b→∞ and the analytical form of the IP. To determine whether QDI provides accurate data fitting, we apply it to orientationally averaged dMRI data from two open source human brain datasets (Dataset 1: Afzali et al. [47], with b‐values in the range 0≤b≤15,000 s mm^−2^; Dataset 2: Tian et al. [48], 0≤b≤17,800 s mm^−2^) and an ex vivo rat brain (Dataset 3: Ingo et al. [49], 0≤b≤22,543 s mm^−2^) and compute novel signal IP maps. We also demonstrate that QDI could be applied to dMRI data acquired in clinically feasible time by comparison of the full dMRI acquisition for dataset 1 (12 *b*‐values, 0≤b≤15,000 s mm^−2^) to a data subset with fewer b‐values (4 *b‐*values, 0≤b≤15,000 s mm^−2^). Finally, we investigate the effect of increasing diffusion time on QDI parameters in the ex vivo rat data.

## 2 Theory

### 2.1 The QDI Functional Form

We begin with Callaghan's classical result for directionally averaged dMRI signal from a cylinder, which is the conventional equation for diffusion in an impermeable intraneurite space and has a negative power law exponent of α=0.5 [14],

(1) $$\;\frac{S_{b}}{S_{0}}=\exp\left(-D_{⊥}b\right)\sqrt{\frac{\pi }{4}}\frac{\mathrm{erf}\left(\sqrt{b\left(D_{\|}-D_{⊥}\right)}\right)}{\sqrt{b\left(D_{\|}-D_{⊥}\right)}},$$

where $S_{b}$ is the signal at a given b‐value (in s mm^−2^), $S_{0}$ is the signal at b=0 s mm^−2^, $D_{\|}$ is the diffusion coefficient (in mm^2^ s^−1^) along the cylinder, and $D_{⊥}$ is the diffusion coefficient (in mm^2^ s^−1^) across the cylinder. Equation (1) describes the behaviour of diffusion in well‐organised axons, where the myelin sheath is assumed to provide an impermeable barrier to water diffusion. The quasi‐diffusion analogue of Equation (1) when α=0.5 is such that the signal decay is given by [17],

(2) $$E_{1/2}\left(-\sqrt{D_{1,2}b}\right)=\exp\left(-D_{1,2}b\right)\left(1-\mathrm{erf}\left(\sqrt{D_{1,2}b}\right)\right),$$

which is analogous to the conventional equation for diffusion in an impermeable intraneurite space [44]. The QDI technique parameterises dMRI signal by the quasi‐diffusion coefficient, $D_{1,2}$ (in mm^2^ s^−1^), and the fractional exponent α that indicates the negative power law behaviour as b→∞. Gaussian diffusion is present when α=1 and non‐Gaussian diffusion when 0<α<1. The characteristic equation of the QDI signal decay is given by the MLF [33, 34], which is a generalisation of the exponential function [50],

(3) $$\frac{S_{b}}{S_{0}}=E_{\alpha }\left(-{\left(D_{1,2}b\right)}^{\alpha }\right)=\sum_{k=0}^{\infty }\frac{{\left(-1\right)}^{k}{\left(D_{1,2}b\right)}^{\alpha k}}{\Gamma \left(\mathrm{\alpha k}+1\right)},$$

where $\Gamma \left(\cdot \right)$ is the gamma function. α provides a measure of tissue heterogeneity, such that lower α corresponds to increased heterogeneity [33, 34, 35]. Equation (3) is a convenient representation of dMRI signal attenuation, as it interpolates between a stretched exponential at low b‐values and a negative power law at high b‐values, both with exponent α. The use of the MLF is the simplest method for generalising such a transition to negative power law decay and is sensitive to tissue heterogeneity in healthy and pathological tissue.

Equation (3) has asymptotic properties in the low and high b‐value regimes according to,

(4) $$E_{\alpha }\left(-{\left(D_{1,2}b\right)}^{\alpha }\right)\sim \left\{\begin{array}{c} \exp\left[-\frac{{\left(D_{1,2}b\right)}^{\alpha }}{\Gamma \left(\alpha +1\right)}\right]\;\;\;\;\;\;\;\;\;\;\;\;\;\;\;\;\;\;\;\;\;\;\;b\to 0,\\ \;\frac{{\left(D_{1,2}b\right)}^{-\alpha }}{\Gamma \left(1-\alpha \right)}=\frac{\sin\left(\alpha \pi \right)}{\pi }\;\frac{\Gamma \left(\alpha \right)}{{\left(D_{1,2}b\right)}^{\alpha }},\;b\to \infty . \end{array}\right.$$

The transition between the stretched exponential and negative power law regimes is via an IP at a particular b‐value. For a non‐Gaussian exponent α (i.e., α≠1), as b→0, the quasi‐diffusion functional form tends to a stretched exponential for which the derivative $\frac{\partial \log\left(S\right)}{\partial b}\to -\infty$. This is in contrast to the finite gradient indicated by the conventional Gaussian diffusion model, the diffusional kurtosis (second‐order cumulant) representation and signal models such as Neurite Orientation Dispersion and Density Imaging (NODDI) [51], SANDI [22] and NEXI [23]. When α=1, the MLF reduces to a monoexponential Gaussian form, and the derivative behaves as expected. For α≠1, the functional form is a representation of the signal attenuation of the ensemble diffusion process within a heterogeneous tissue environment for a wide range of b‐values; QDI is applicable to all healthy and pathological tissue voxels.

The QDI functional form (Equation 3) is also the solution of the quasi‐diffusion fractional Fokker–Planck equation given by [33, 34] as,
(5)

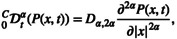

where $P\left(x,t\right)$ is the diffusion propagator, 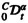 is the Caputo fractional derivative (which is the αth fractional order time derivative for 0<α<1), $\partial^{2\alpha }/\partial {\left|x\right|}^{2\alpha }$ is the Riesz fractional derivative (which is the $\left(2\alpha \right)\mathrm{th}$ fractional order space derivative for 0<2α<2) and $D_{\alpha ,2\alpha }$ is the normal effective diffusion coefficient in units of mm^2α^ s^−α^. The quasi‐diffusion coefficient in units of mm^2^ s^−1^ can be recovered as $D_{\alpha ,2\alpha }=D_{1,2}^{\alpha }={\left(D_{1,2}\right)}^{\alpha }$. The QDI signal attenuation as parameterised by the normal effective diffusion coefficient contains a continuous distribution of diffusion coefficients within a voxel [34]. There is only a single Gaussian ADC when α=1. Conceptually, as α decreases, the diffusion environment becomes more heterogeneous, and the distribution of constituent diffusion coefficients in the QDI signal becomes broader [34]. Although QDI provides a normal effective diffusion coefficient that increases linearly with time, the technique is based on a model of anomalous diffusion dynamics where step lengths have finite mean but infinite variance, and waiting times have infinite mean in the CTRW.

### 2.2 Determining the b‐Value of the Inflection Point

The b‐value of the IP may be calculated by estimating the zero crossing of the second derivative of the logarithm of Equation (3) with respect to $\ln\left(b\right)$. Analytical derivations are provided in the Supporting Information, but in brief, the mathematical analysis requires the single‐parameter MLF (Equation 3) to be written as a two‐parameter MLF [51, 52], which is given by,

(6) $$E_{\alpha ,\gamma }\left(z\right)=\sum_{k=0}^{\infty }\frac{z^{k}}{\Gamma \left(\mathrm{\alpha k}+\gamma \right)}\;z\in \mathbb{C},\alpha >0\in \mathbb{R},\gamma \in \mathbb{C},$$

with γ=1 defining the single‐parameter MLF. To simplify notation in this section we substitute $\lambda =D_{1,2}^{\alpha }$ for the normal effective diffusion coefficient. The derivatives of,

(7) $$y=\ln\left(E_{\alpha ,1}\left(-\lambda b^{\alpha }\right)\right)$$

may be obtained using the equation for the derivative of the two‐parameter MLF [51, 52],

(8) $$\frac{d}{\mathrm{dz}}\left[E_{\alpha ,\gamma }\left(z\right)\right]=\frac{E_{\alpha ,\gamma -1}\left(z\right)-\left(\gamma -1\right)E_{\alpha ,\gamma }\left(z\right)}{\mathrm{\alpha z}},$$

by application of the chain and quotient rules for differentiation. Throughout this paper we consider the function $\ln\left(b/b_{0}\right)$ to be defined for $b_{0}=1$ s mm^−2^ and hence equivalent to a unitless $\ln\left(b\right)$. The analytical forms of the first and second derivatives with respect to $\ln\left(b\right)$ are,

(9) $$\frac{\mathrm{dy}}{d\left(\ln\left(b\right)\right)}=\frac{E_{\alpha ,0}\left(-\lambda b^{\alpha }\right)}{E_{\alpha ,1}\left(-\lambda b^{\alpha }\right)}$$

and

(10) $$\begin{array}{c} \frac{d^{2}y}{d{\left(\ln\left(b\right)\right)}^{2}}=\frac{\left(E_{\alpha ,-1}\left(-\lambda b^{\alpha }\right)+E_{\alpha ,0}\left(-\lambda b^{\alpha }\right)\right)E_{\alpha ,1}\left(-\lambda b^{\alpha }\right)-{\left(E_{\alpha ,0}\left(-\lambda b^{\alpha }\right)\right)}^{2}}{{\left(E_{\alpha ,1}\left(-\lambda b^{\alpha }\right)\right)}^{2}}, \end{array}$$

with the b‐value of the IP occurring when $\frac{d^{2}y}{d{\left(\ln\left(b\right)\right)}^{2}}=0$ such that

(11) $$\left(E_{\alpha ,-1}\left(-\lambda b^{\alpha }\right)+E_{\alpha ,0}\left(-\lambda b^{\alpha }\right)\right)E_{\alpha ,1}\left(-\lambda b^{\alpha }\right)-{\left(E_{\alpha ,0}\left(-\lambda b^{\alpha }\right)\right)}^{2}=0.$$

## 3 Methods

### 3.1 Image Acquisition

Three ultra‐high *b*‐value dMRI datasets were analysed: two human participant open‐source datasets and one rat. Informed consent was obtained for human participants. All sample preparation and image acquisition parameters are presented in abbreviated form with full information in Afzali et al. [47] (Dataset 1), Tian et al. [48] (Dataset 2) and Ingo et al. [49] (Dataset 3).

#### 3.1.1 Dataset 1

An open‐source dataset of whole brain dMRI acquired from a single healthy participant [47]. dMRI data were acquired using a 3T Connectom MR imaging system with maximum gradient strength 300 mT m^−1^ (Siemens Healthineers, Erlangen, Germany, https://www.siemens‐healthineers.com/). Sixty‐six axial slices were acquired using a 2D single refocussed pulsed gradient spin echo (srPGSE) sequence with acquisition parameters: TE/TR = 55/4000 ms, δ/Δ = 12/23 ms at 2 mm isotropic voxel resolution. Six b=0 s mm^−2^ images and 11 diffusion‐sensitised images were acquired at $\begin{array}{c} \begin{array}{c} b=\left\{400,800,1200,2000,3000,4000,6000,8000,\right. \end{array}\\ \begin{array}{c} \left.10000,12000,15000\right\} \end{array} \end{array}$ s mm^−2^ with gradient strengths $\begin{array}{c} g=\\ \left\{45,64,78,101,124,143,175,201,226,248,277\right\} \end{array}$ mT m^−1^ applied in 16,16,21,31,21,21,31,31,31,31 and 46 diffusion gradient directions. All diffusion gradient directions were uniformly distributed on a sphere. Acquisition time was 20 min and 8 s. Image preprocessing is described in Afzali et al. [47].

#### 3.1.2 Dataset 2

An open‐source dataset of whole brain dMRI acquired from a single participant (Subject 001) [48]. Real and magnitude dMRI data were acquired using a 3T Siemens Connectom MR imaging system with maximum gradient strength 300 mT m^−1^. Sixty‐six sagittal slices were acquired using a srPGSE sequence: TE/TR = 77/3800 ms, δ/Δ = 8/49 ms at 2 mm isotropic voxel resolution. Fifty b=0 s mm^−2^ images and eight diffusion‐sensitised images were acquired at $\begin{array}{c} \begin{array}{c} b=\left\{200,950,2300,4250,6750,9850,13500,17800\right\} \end{array} \end{array}$ s mm^−2^ with gradient strengths $g=\left\{31,67,104,142,178,215,253,290\right\}$ mT m^−1^ applied in 32 diffusion gradient directions for b<2400 s mm^−2^ and 64 directions for b≥2400 s mm^−2^. All diffusion gradients were uniformly distributed on a sphere. Magnitude dMRI data were analysed in our study. Acquisition time was 55 min. Image preprocessing is described in Tian et al. [48].

#### 3.1.3 Dataset 3

A single dMRI slice of an ex vivo healthy fixed rat brain [49]. Prior to imaging, the rat brain was soaked overnight in phosphate buffered saline. The brain was placed in a 20 mm imaging tube that was filled with Fluorinert and secured with a magnetic susceptibility matched plug to minimise vibrational movement due to the pulsed gradients. Scanning was performed on a Bruker spectrometer at 750 MHz (17.6 T, 89 mm bore) with the anterior–posterior brain orientation along the main *B*_0_ field (*z*‐axis) the superior–inferior along the *x*‐axis, and left–right along the *y*‐axis. Pulsed gradient stimulated echo (PGSTE) images were acquired: TE/TR = 28/2000 ms, δ/Δ = 3.5/17.5 ms, with mixing time 8 ms, in‐plane resolution 190 μm and slice thickness 1 mm. Diffusion‐sensitised images were acquired at eleven *b*‐values $\begin{array}{c} \begin{array}{c} b=\left\{0,1000,2000,3000,6000,9000,12000,15000,18000,\right. \end{array}\\ \begin{array}{c} \left.21000,25000\right\} \end{array} \end{array}$ s mm^−2^ with gradient strengths $\begin{array}{c} g_{17.5}=\left\{0,264,\right.\\ \left.374,458,647,793,915,1023,1121,1211,1321\right\} \end{array}$ mT m^−1^ applied along the three perpendicular scanner axes. dMRI were acquired twice to improve signal to noise ratios. Variable TR data were acquired to correct for T1 relaxation effects (TE = 12.5 ms, TR = 300 to 3600 ms in increments of 300 ms).

A similar dMRI protocol was acquired for Δ of 17.5, 50, 100 and 200 ms with a diffusion gradient applied along the *y*‐axis. Corresponding diffusion gradient strengths were $\begin{array}{c} g_{50}=\left\{0,153,\right.\\ \left.216,265,374,459,529,592,648,700,764\right\} \end{array}g_{50}=\left\{0,153,216,265,374,459,529,592,648,700,764\right\}$, $\begin{array}{c} g_{100}=\left\{0,107,152,186,\right.\\ \left.263,322,372,416,456,492,537\right\} \end{array}g_{100}=\left\{0,107,152,186,263,322,372,416,456,492,537\right\}$ and $\begin{array}{c} g_{200}=\left\{0,76,107,131,186,\right.\\ \left.227,262,293,347,379\right\} \end{array}g_{200}=\left\{0,76,107,131,186,227,262,293,347,379\right\}$ mT m^−1^ with mixing times 40, 90 and 190 ms, respectively.

### 3.2 Image Pre‐Processing

Magnitude dMRI data were corrected for background Rician noise properties estimated from regions of interest (ROIs) containing no tissue signal. An ROI in ventricular cerebrospinal fluid (CSF) at *b* = 17,800 s mm^−2^ was used for Dataset 2, and all voxels outside the tissue sample was used for Dataset 3. The standard deviation of Gaussian noise, *σ*, was estimated by subtracting dMRI signal in each gradient direction from every other and calculating the standard deviation within the ROI. Mean Rician noise, $\mu_{R}$, was estimated by [53],

(12) $$\mu_{R}=\sigma \sqrt{\pi /2},$$

and Rician noise correction was performed using [53],

(13) $$S^{2}=S_{C}^{2}+\mu_{R}^{2},$$

where S is the acquired dMRI signal and $S_{C}$ is dMRI signal corrected for Rician noise.

### 3.3 Model Fitting

Orientationally averaged signal (when available) was used in data fitting. To provide balanced weighting of the residuals across four orders of magnitude of signal decay and to enable robust fitting of the signal tail, the data were fitted in the natural logarithmic space using,

(14) $$\ln\left(\frac{S_{b}}{S_{0}}\right)=\ln\left(E_{\alpha }\left(-{\left(D_{1,2}b\right)}^{\alpha }\right)\right).$$

Data fitting was performed to estimate $D_{1,2}$ (in mm^2^ s^−1^) and α (unitless) using the trust‐region‐reflective algorithm in Matlab (https://www.mathworks.com). The MLF was computed using Garrappa's numerical algorithm [54, 55].

The gradient (first derivative) of the natural logarithm of $E_{\alpha }\left(-{\left(D_{1,2}b\right)}^{\alpha }\right)$ with respect to $\ln\left(b\right)$ was calculated using Equation (9). b‐value IPs were calculated numerically by estimating the zero crossing of Equation (11) for $0<\ln\left(b\right)<50$ in steps of 0.001. The maximum value of $\ln\left(50\right)$ was chosen to ensure that *b*‐value IPs could be calculated for low $D_{1,2}$ as α→0.5. The two parameter MLF was estimated numerically [54, 55]. To enable rapid estimation of *b*‐value IPs from $D_{1,2}$ and α a lookup table of *b*‐value IPs was constructed for $0.001\leq D_{1,2}\leq 3\times 10^{-3}$ mm^2^ s^−1^ and 0.5≤α<1.0.

### 3.4 Image Analysis

#### 3.4.1 Dataset 1

To assess the QDI functional form of dMRI signal attenuation, $D_{1,2}$ and α were estimated between b=0 s mm^−2^ and maximum b‐values ($b_{\max}$) in the range $1200\leq b_{\max}\leq 15,000$ s mm^−2^. To enable computation of tissue properties, the brainstem and cerebellum were manually removed and tissue segmentation (of GM, WM and CSF) was performed using the FSL fast technique [56] on input of $D_{1,2}$, α and IP maps that were estimated across the full b‐value range ($b_{\max}=15,000$ s mm^−2^). Median and quartile values for $D_{1,2}$, α and IPs were calculated in cerebral GM and WM ROIs for each subject.

Quality of model fitting to observed signal was computed for the natural logarithm of the estimated quasi‐diffusion signal attenuation (determined by data fitting across each $b_{\max}$ range, $0\leq b\leq b_{\max}$ s mm^−2^ for $1200\leq b_{\max}\leq 15,000$ s mm^−2^) in comparison to the natural logarithm of the observed normalised signal attenuation over the full b‐value range (0≤b≤15,000 s mm^−2^) using mean squared error (MSE). Median and quartiles of the MSE were calculated in cerebral GM and WM voxels.

To investigate whether quasi‐diffusion parameters can be accurately and reliably estimated from dMRI acquired in clinically feasible time (defined here as approximately 5 min), $D_{1,2}$, α and IPs were estimated from $b_{\text{short}}=\left\{0,1200,4000,15000\right\}$ s mm^−2^ data (acquisition time 6 min and 16 s, 31% of the full dMRI acquisition time). b‐values were chosen to provide observed data over three decades of the natural logarithm of the b‐value, with two b‐values prior to expected IPs and one afterwards. Measurement bias was calculated for $D_{1,2}$, α and IP measures as the mean of the voxel‐wise estimates for the short acquisition measures minus those for the full acquisition (referred to here as the voxel‐wise difference). Measurement uncertainty was calculated as the standard deviation of the voxel‐wise difference. Intraclass correlation coefficients (ICCs) were also calculated. Bias, uncertainty and ICC were calculated across all GM and WM tissue voxels for $D_{1,2}$, α and IP.

#### 3.4.2 Datasets 2 and 3

To further demonstrate the utility of QDI, it was applied to the full b‐value range of b‐values for Datasets 2 (0≤b≤17,800 s mm^−2^) and 3 (0≤b≤25,000 s mm^−2^). Median and quartiles of $D_{1,2}$, α and IP were calculated in cortical GM, WM and corpus callosum (CC) ROIs.

## 4 Results

### 4.1 Quasi‐Diffusion Signal Behaviour

**FIGURE 1:**
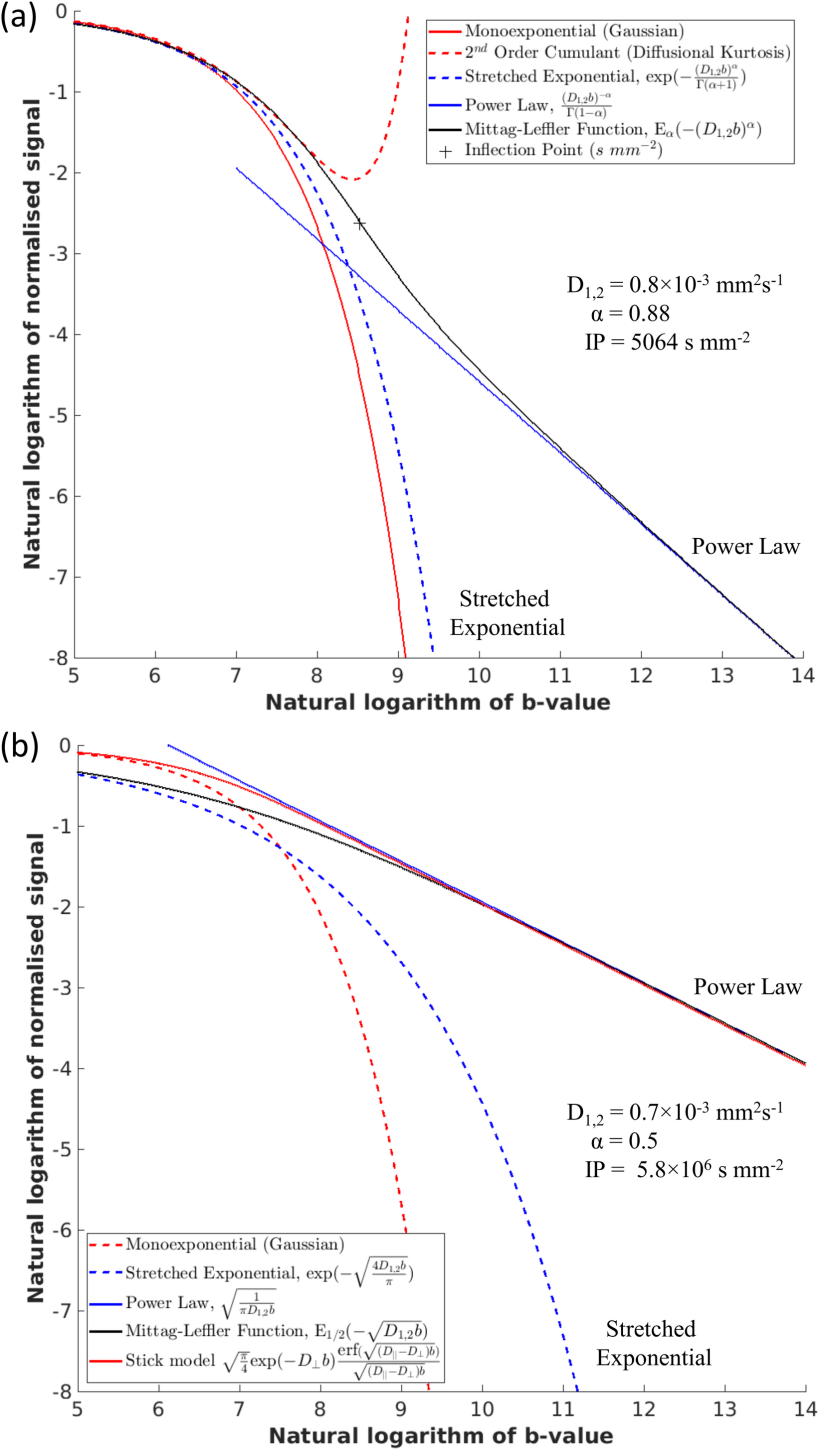
Predicted signal attenuation for the quasi‐diffusion model. The stretched Mittag–Leffler function is illustrated in the logarithmic space of $\ln\left(E_{\alpha }\left(-{\left(D_{1,2}b\right)}^{\alpha }\right)\right)$ against $\ln\left(b\right)$. This shows its transition between a stretched exponential at low b‐values and a negative power law at high b‐values. The transition is marked by an inflection point (cross) in the logarithmic space. Predicted signal attenuation is shown for a typical grey matter voxel (a) with $D_{1,2}=0.8\times 10^{-3}$ mm^2^ s^−1^ and α=0.88 with Gaussian and diffusional kurtosis approximations shown. Signal attenuation is also shown for a white matter voxel (b) with $D_{1,2}=0.7\times 10^{-3}$ mm^2^ s^−1^ and α=0.5 in comparison to the behaviour of the intraneurite stick model.

A feature of signal attenuation described by quasi‐diffusion is a transition between stretched exponential and negative power law regimes via an IP in the logarithmic space. Figure 1a shows this behaviour for predicted quasi‐diffusion signal attenuation in a representative GM voxel given by $D_{1,2}=0.8\times 10^{-3}$ mm^2^ s^−1^ and α=0.88 on a log–log plot. The illustrated stretched exponential and power law behaviour characterise the limits as b→0 and b→∞, respectively. Gaussian (free diffusion) and diffusional kurtosis representations do not capture the power law behaviour. Figure 1b shows quasi‐diffusion attenuation in a WM voxel with $D_{1,2}=0.8\times 10^{-3}$ mm^2^ s^−1^ and α=0.5 in comparison to the intraneurite stick model. Both models identify the negative power law behaviour, but differences are apparent as b→0 where the stick model follows Gaussian signal attenuation.

**FIGURE 2:**
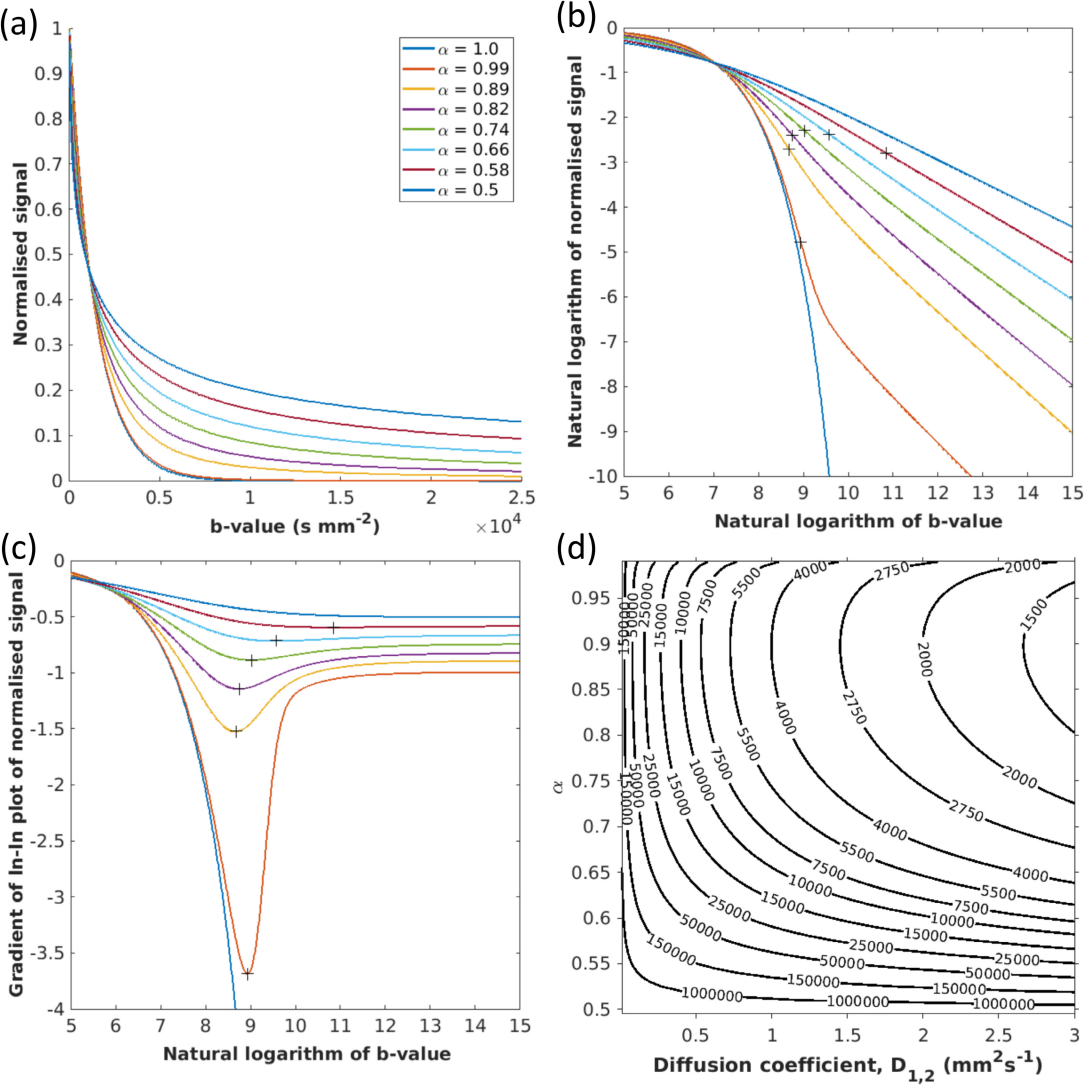
Predicted signal attenuation for the quasi‐diffusion model when $D_{1,2}=0.7\times 10^{-3}$ mm^2^ s^−1^ and 0.5≤α≤1. Graphs show (a) the normalised signal attenuation for the family of curves, (b) signal attenuation in a log–log plot of the natural logarithm, and (c) the gradient of the log–log plot. At high b‐values, the gradient tends to a value of −α. Inflection points are shown as crosses in (b) and (c). The contour map (d) shows the b‐values at which inflection points occur for different combinations of $D_{1,2}$ ($0\leq D_{1,2}\leq 3\times 10^{-3}$ mm^2^ s^−1^) and α (0.5≤α<1).

Figure 2 shows predicted quasi‐diffusion signal attenuation for $D_{1,2}=0.7\times 10^{-3}$ mm^2^ s^−1^ with 0.5≤α≤1 (Figure 2a) and IPs in the logarithmic space (Figure 2b). Figure 2c shows the first derivative, or slope, in the log space. Signal inflections become increasing shallow at increasing b‐values for decreasing values of α, and after inflection, the signal enters a negative power law signal decay where the gradient tends to −α. The b‐value of the IP is shown as a contour map in Figure 2d as a function of $D_{1,2}$ for $0<D_{1,2}\leq 3\times 10^{-3}$ mm^2^ s^−1^ and α for 0.5≤α≤1. Contours are smooth and indicate that lower $D_{1,2}$ and/or α leads to higher b‐value IPs, with higher $D_{1,2}$ and/or α leading to lower b‐value IPs. Higher b‐value IPs are also observed as α→1. Specifically, as $D_{1,2}$ increases the b‐value of the IPs decrease for a given α, with the contrary for decreasing $D_{1,2}$.

### 4.2 Human and rat Brain Data

#### 4.2.1 Dataset 1

**FIGURE 3:**
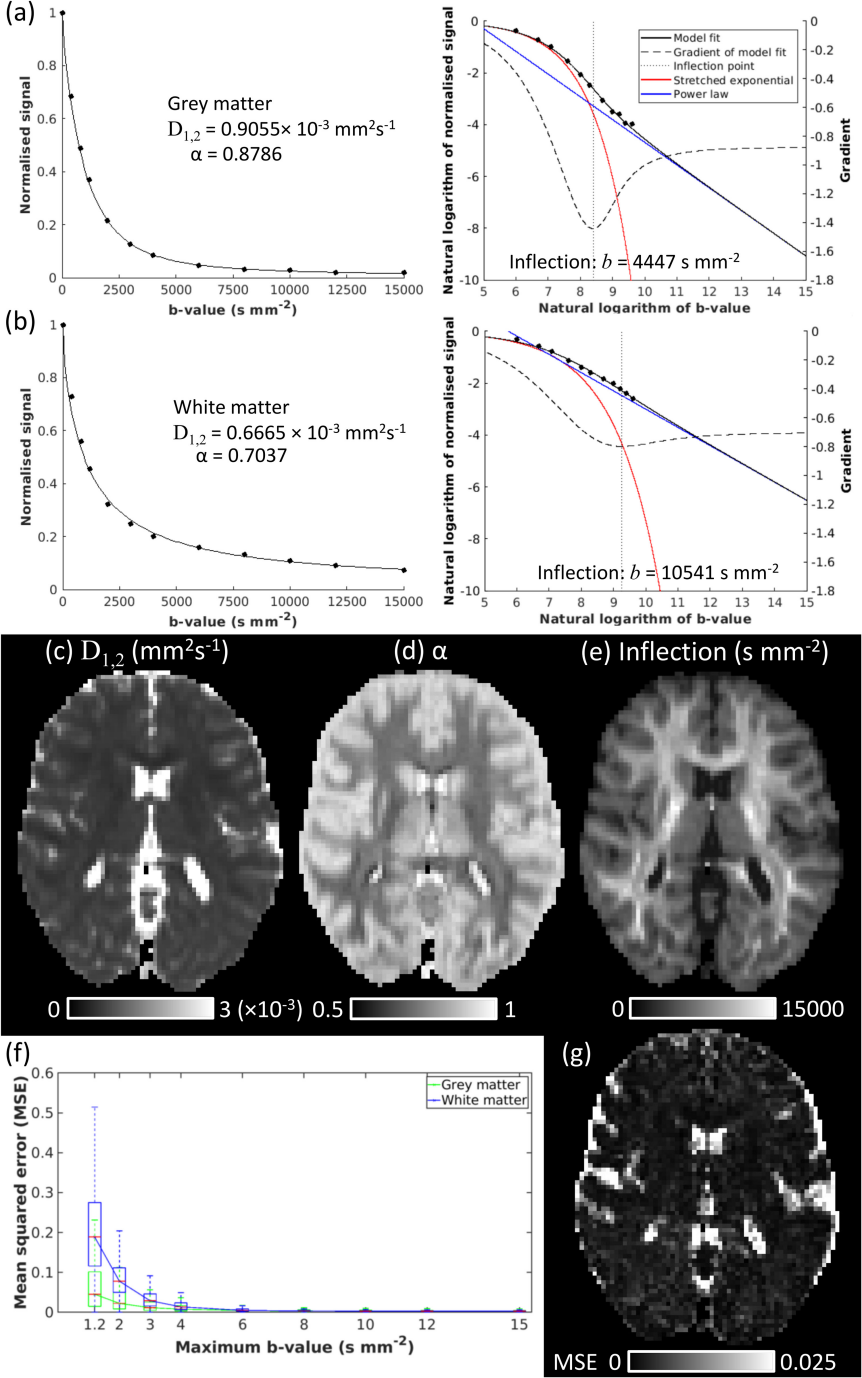
Signal attenuation for representative (a) grey matter and (b) white matter voxels is shown with axial slices of (c) $D_{1,2}$, (d) α, and (e) IP maps for in vivo human Dataset 1. Signal attenuation is shown for normalised signal (left graphs) and on a log–log plot (right graphs). The quasi‐diffusion model fit (black line) and estimated $D_{1,2}$ and α values are indicated on the graphs. The *b*‐value of the IP (dotted black line), the stretched exponential (red) and power law (blue) limits, and the gradient of the quasi‐diffusion signal (dashed black line) are shown in the log–log plots. (f) Box and whisker plots for median values of mean squared error (MSE) for QDI fitting across grey matter (green) and white matter (blue) voxels against increasing maximum *b*‐value ($1200\leq b_{\max}\leq 15,000$ s mm^−2^). (g) An axial slice of the MSE map for $b_{\max}=15,000$ s mm^−2^.

Figure 3 shows excellent QDI fits (black line) to dMRI signal within representative GM (Figure 3a) and WM (Figure 3b) voxels for 0≤b≤15,000 s mm^−2^ with the stretched exponential (red) and power law (blue) showing the model fit as b→0 and b→∞, respectively. IPs (dotted black line) occur before the maximum b‐value (b=15,000 s mm^−2^) in each voxel. The gradient of the model fit (dashed black line) shows that modelled signal changes substantially around the IP and the negative power law exponent, α, is reached at ultra‐high *b*‐values (right axis).

**FIGURE 4:**
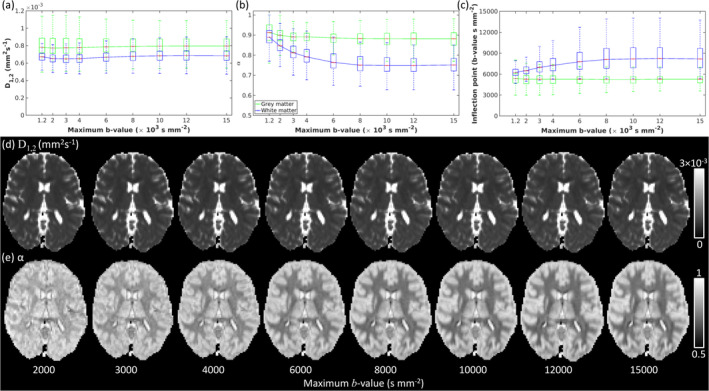
Box and whisker plots are shown of estimated (a) $D_{1,2}$, (b) α, and (c) IP with increasing maximum *b*‐value for grey (green) and white matter (blue). Maximum *b*‐value ranges are shown between $1200\leq b_{\max}\leq 15,000$ s mm^−2^. Axial slices for (d) $D_{1,2}$ and (e) α maps are also shown when computed for different maximum *b*‐values ($2000\leq b_{\max}\leq 15,000$ s mm^−2^).

Maps of $D_{1,2}$ (Figure 3c), α (Figure 3d) and IP (Figure 3e) calculated for the range 0≤b≤15,000 s mm^−2^ demonstrate tissue specific values and high tissue contrast in α and IP maps. Figure 4f plots the MSE in quasi‐diffusion model fitting for each $b_{\max}$ range, $0\leq b\leq b_{\max}$ s mm^−2^ (for $1200\leq b_{\max}\leq 15,000$ s mm^−2^) compared to observed signal over the full b‐value range (0≤b≤15,000 s mm^−2^). The quality of model fitting increases as $b_{\max}$ increases with highly accurate fitting achieved for $b_{\max}\geq 6000$ s mm^−2^ in brain tissue (Figure 3g).

Tissue $D_{1,2}$ distributions (Figure 4a) overlapped with higher values in GM (median $D_{1,2}=0.796\times 10^{-3}$ mm^2^ s^−1^) than WM (median $D_{1,2}=0.684\times 10^{-3}$ mm^2^ s^−1^) and greater interquartile range due to partial volume effects at the GM/CSF boundary. Tissue α distributions (Figure 4b) revealed higher values in GM (median α=0.882) than WM (median α=0.751) and similar interquartile ranges. The range of α extended towards Gaussian properties in GM but did not extend to α=0.5 in WM. Signal IP distributions (Figure 4c) had lower values in GM (median IP=5271 s mm^−2^) than WM (median IP=8185 s mm^−2^) with greater positive skew observed for WM as b→15,000 s mm^−2^. Figure 4a,b,d,e shows that $D_{1,2}$ and α estimates tend to stable values as $b_{\max}$ increases, an effect related to the quality of model fitting (Figure 3f,g), with α estimates tending towards a Gaussian diffusion exponent of 1 at lower $b_{\max}$ values. Fitted QDI parameters stabilise when $b_{\max}$ approaches or exceeds the IP, which is at lower b‐values in GM (b≥3000 s mm^−2^) than WM (b≥6000 s mm^−2^).

**FIGURE 5:**
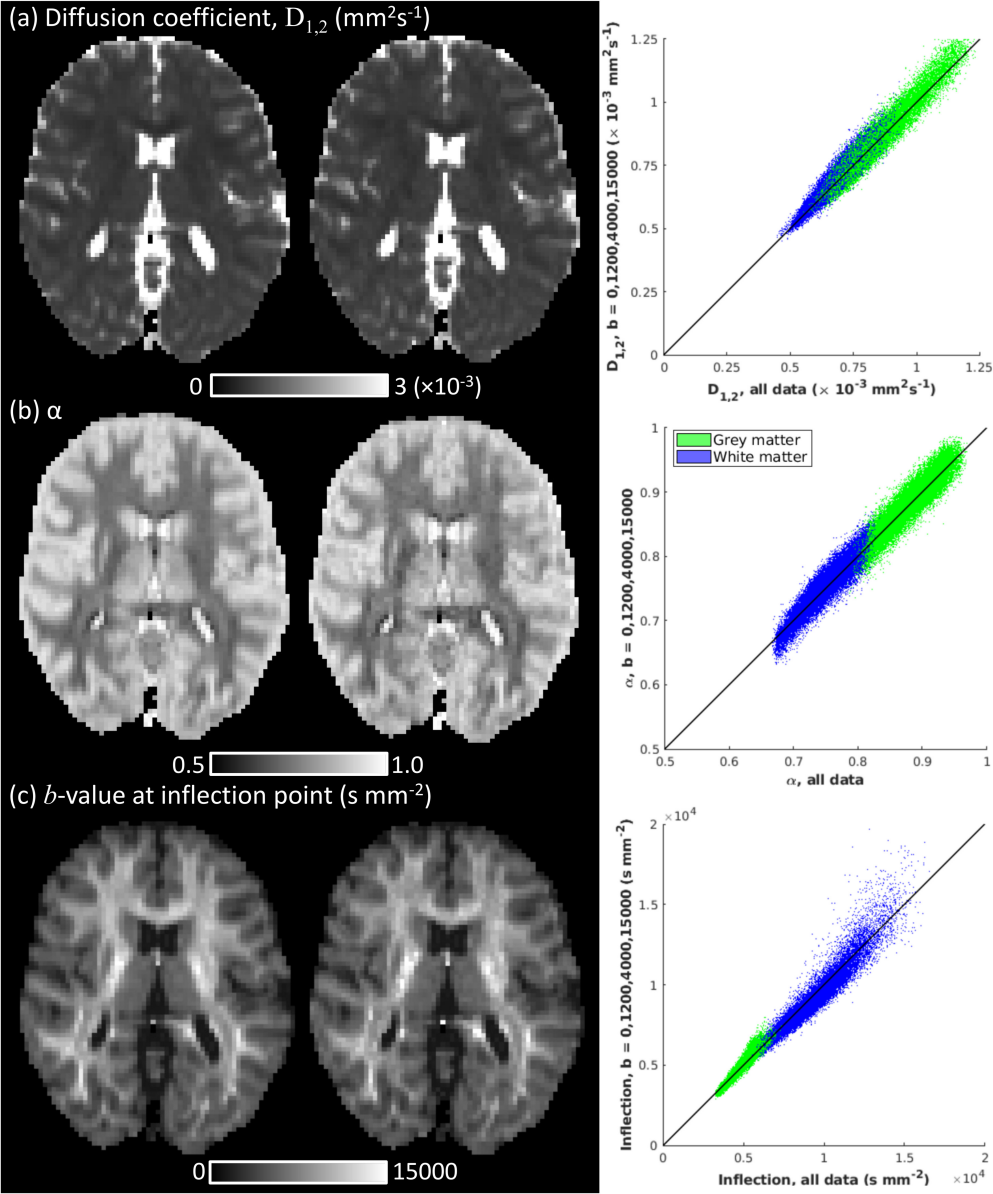
Comparison of $D_{1,2}$, α, and IP calculated from in vivo human Dataset 1 for the full acquisition (12 *b‐*values, 0≤b≤15,000 s mm^−2^) and a subset of the data (4 *b*‐values, $b=\left\{0,1200,4000,15000\right\}$ s mm^−2^). Axial slices of (a) $D_{1,2}$, (b) α, and (c) IP maps are illustrated for the full acquisition (left column) and data subset (middle column). Graphs (right column) show scatter plots of voxel values estimated for $D_{1,2}$, α, and IP for the data subset against the full acquisition in grey (green) and white (blue) matter.

Figure 5 demonstrates that $D_{1,2}$, α and IP estimation are highly accurate, although slightly noisier, when fitted from $b_{\text{short}}=\left\{0,1200,4000,15000\right\}$ s mm^−2^ compared to all data (0≤b≤15,000 s mm^−2^). Measurement bias was small, approximately 1.1% of median $D_{1,2}$ across brain tissue ($\text{bias}=-0.008\pm 0.031\times 10^{-3}$ mm^2^ s^−1^), approximately 0.3% of median α (bias=0.002±0.014), and approximately 1.4% of median IP (bias=94±388 s mm^−2^). ICCs were extremely high across brain tissue for $D_{1,2}$ (ICC=0.970), α (ICC=0.982) and IP (ICC=0.985) indicating that a subsample of the acquired dMRI data provides reproducible parameter estimation compared to the full acquisition.

#### 4.2.2 Datasets 2 and 3

**FIGURE 6:**
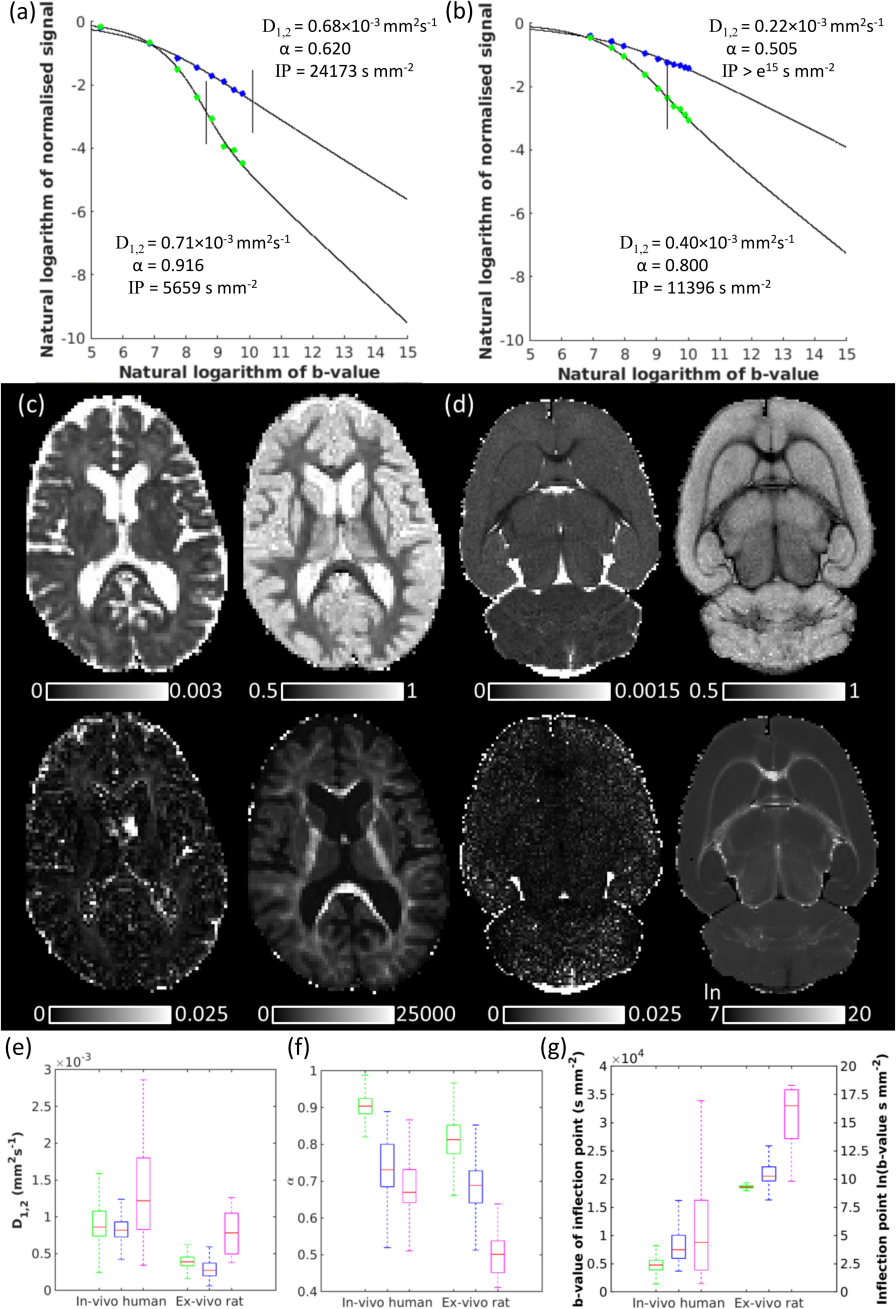
Quasi‐diffusion imaging for in vivo human Dataset 2 (0≤b≤17,800 s mm^−2^) and ex vivo rat Dataset 3 (0≤b≤25,000 s mm^−2^). (a, b) Signal attenuation and QDI fits for representative cortical (green) and midsagittal corpus callosum white matter (blue) voxels for the human and rat data, respectively. IPs are marked on the decay curves by a vertical black line. (c, d) Axial slices (clockwise from top left) of $D_{1,2}$, α, IP, and MSE maps for each dataset, respectively. Box and whisker plots are shown for (e) $D_{1,2}$, (f) α, and (g) IP in grey matter (green), white matter (blue), and corpus callosum (magenta) regions of interest for both datasets.

Figure 6 shows results for the second human participant (0≤b≤17,800 s mm^−2^) and an ex vivo rat (0≤b≤25,000 s mm^−2^). QDI provided excellent fitting to dMRI signal in cortical (green) and CC (blue) voxels (Figure 6,b) and low MSE fitting (Figure 6c,d). Similar tissue contrast (Figure 6c) and parameter distributions (Figure 6e–g) were found for Dataset 2 compared to Dataset 1 (median $D_{1,2}$: GM $0.861\times 10^{-3}$ mm^2^s^−1^, WM $0.819\times 10^{-3}$ mm^2^ s^−1^; median α: GM 0.904, WM 0.731; median IP: GM 4779 s mm^−2^; WM 7469 s mm^−2^). In the CC, median $D_{1,2}$ ($1.219\times 10^{-3}$ mm^2^ s^−1^) was higher due to partial volume effects with CSF, and median α (0.670) was closer to the intraneurite power law. The ex vivo rat data had greater tissue contrast (Figure 6d) and lower median $D_{1,2}$ and α values than in vivo human data ($D_{1,2}$: GM $0.439\times 10^{-3}$ mm^2^ s^−1^, WM $0.368\times 10^{-3}$ mm^2^ s^−1^, Figure 6e; α: GM 0.813, WM 0.689, Figure 6f). A lower median $D_{1,2}$ was found in the CC compared to WM ($0.186\times 10^{-3}$ mm^2^ s^−1^) due to reduced CSF partial volume effects at high image resolution. Median α (0.517) in the CC corresponded to the intraneurite power law. As $D_{1,2}$ and α were lower in the ex vivo rat than in vivo human data, the median IPs were substantially higher (GM 10,915 s mm^−2^, WM 28,427 s mm^−2^, CC 14,707,969 s mm^−2^).

**FIGURE 7:**
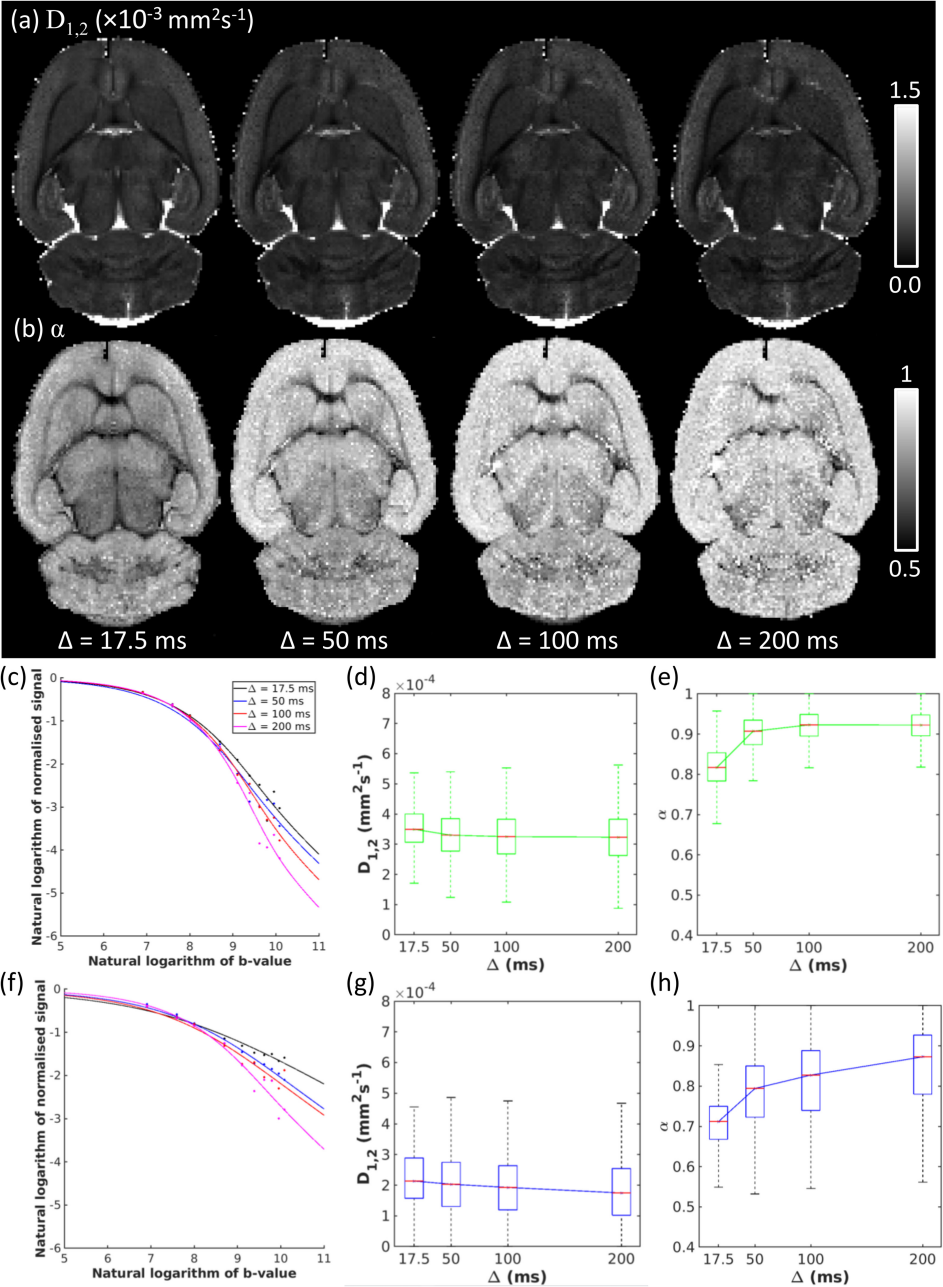
Effect of increasing diffusion time on quasi‐diffusion images for the ex vivo rodent dataset. Axial slices are shown for (a) $D_{1,2}$ and (b) α maps at diffusion times of ∆ = 17.5, 50, 100 and 200 ms with fixed δ=3.5 ms. QDI fits to signal attenuation are presented in log–log plots for representative (c) cortical grey matter and (d) corpus callosum voxels for ∆=17.5 ms (black), ∆=50 ms (blue), ∆=100 ms (red) and ∆=200 ms (magenta). Time‐dependence of $D_{1,2}$ (graphs d and g) and (graphs e and h) are is shown for grey matter (green) and white matter (blue) regions of interest as box and whisker plots.

Figure 7 shows time dependence of $D_{1,2}$ (Figure 7a) and α (Figure 7b) maps for the ex vivo rat data. QDI provided excellent data fitting in representative cortical (Figure 7c) and CC (Figure 7f) voxels despite greater noise at longer diffusion times. Median $D_{1,2}$ decreased as the tortuosity limit was approached in GM (∆=17.5,50,100,200 ms, $0.350,0.330,0.325,0.323\times 10^{-3}$ mm^2^ s^−1^), WM ($0.214,0.203,0.193,0.175\times 10^{-3}$ mm^2^ s^−1^) and CC ($0.342,0.300,0.339,0.317\times 10^{-3}$ mm^2^ s^−1^) as shown in Figure 7d and 7g. Median α increased as power law exponents tended towards a Gaussian tortuosity in GM (0.817,0.907,0.923,0.922) and WM (0.712,0.794,0.827,0.873) with the CC exhibiting slower passage to the tortuosity limit than WM (0.528, 0.588, 0.560, 0.694); see Figure 7e and 7h.

## 5 Discussion

We have shown that the dMRI signal acquired from low *b*‐values to those within the power law regime can be fitted with excellent agreement to the parsimonious QDI functional form. This form includes only two parameters, $D_{1,2}$ and α. Stable parameter estimates were identified upon increasing the maximum b‐value of the fitting range to above the IPs to enable accurate definition of the full decay curve, suggesting that QDI is an accurate representation of dMRI signal in healthy brain tissue. Furthermore, QDI parameters computed from a four b‐value acquisition that samples only one data point beyond the IP are accurate when compared to the full acquisition (12 b‐values), indicating that QDI measurements may be estimated from dMRI acquired in clinically feasible times. Time dependence of QDI parameters has also been demonstrated.

QDI provides a simple representation for signal attenuation that fits well to data and identifies a transition to an experimentally observed power law behaviour. Our results show excellent data fits by QDI across b‐values (up to max b=25,000 s mm^−2^), and the technique has recently been shown to fit data acquired perpendicular to recently deceased mouse spinal cord (max b=858,022 s mm^−2^, δ/∆=11/400 ms) [46]. Many dMRI signal representations and models cannot define this transition and provide signal representation below the *b*‐value IP, including the monoexponential, biexponential [57], stretched exponential [7, 8, 58], second‐order cumulant representation (diffusional kurtosis) [5, 6] and diffusion models with characteristic equations given by a stretched exponential (i.e., space‐fractional superdiffusion [9], fractional motion [10, 11] and transient subdiffusion and superdiffusion [59, 60]).

The ability of QDI to define an experimentally observed power law is in contrast to other signal representation techniques and tissue compartment models that have not explicitly demonstrated this effect despite estimating greater numbers of parameters (i.e., higher order cumulant functions [15, 61], mean apparent propagator imaging [62], q‐space diffusion propagator imaging [63], diffusion spectrum imaging [64], Laplacian eigenfunctions [65, 66], DBSI [67], NODDI [68] and VERDICT [69]). For instance, as more tensors are added to tensor distribution models, the signal attenuation tends to a negative power law, but no signal inflection is observed [70, 71]. Furthermore, a recent extension to the NODDI technique has included an impermeable intraneurite stick compartment [72], which potentially enables modelled signal to include an IP and transition to a negative power law at high *b*‐values. Nevertheless, a limitation of each of these techniques is that a greater number of fitting parameters risks problems of parameter degeneracy and high sensitivity to effects of noise on parameter estimation [73].

Stochastic models with characteristic equations given by a MLF, such as general CTRW diffusion [45, 49, 74] and time‐fractional subdiffusion [45, 75, 76], will identify transition between stretched exponential and power law behaviour via a signal IP. However, QDI is unique in being based on a stochastic model of anomalous diffusion with a normal effective diffusion that is neither superdiffusive nor subdiffusive [33, 34]. Afzali et al. [47] have demonstrated transition to the localisation regime using a higher order cumulant expansion but this involves estimation of five parameters that do not have clear mathematical or physical meaning. GM tissue compartment models [22, 23, 24] can identify transition from Gaussian to power law regimes via a signal IP as shown in experimental data when plotted against $1/\sqrt{b}$. This is due to either assumption of an impermeable intraneurite stick compartment [22, 24] or the inclusion of a Kärger model [77] to quantify cell membrane permeability [23, 24], which subtly alters Gaussian phase approximation assumptions within the modelled signal. In QDI, the α exponent does not transition from a Gaussian (α=1) at b=0 s mm^−2^ to an intraneurite power law scaling of $1/\sqrt{b}$ (α=0.5) as b→∞. Despite this, we have shown that the QDI functional form provides an excellent representation of signal across a large range of b‐values, diffusion gradient durations and diffusion times.

Negative power law exponents estimated by QDI in human GM of α≈0.9 are similar to previously reported exponents [12, 33, 35, 45]. Olesen et al. [24] have shown in GM simulation studies that the intraneurite power law is obscured by properties of the soma and an *apparent* power law is observed in neurites plus soma, which increases with δ to 0.8 at δ/∆=9/30 ms. Olesen et al. [24] suggest this effect is caused by substantial exchange between soma and the extracellular space, non‐negligible neurite exchange and a small population of myelinated impermeable axons. Furthermore, they investigated whether intraneurite power laws could be observed in fixed rat brain at 16.4 T for data acquired at 37°C at short diffusion times and found dMRI signal attenuation to be well approximated by eSANDIX for δ/∆=4/16 ms with an intraneurite power law observed for b‐values > 25,000 s mm^−2^. Our fixed brain rat data at room temperature revealed α≈0.8 in GM at a similar diffusion time (δ/∆=3.5/17.5 ms) but with b‐values ≤ 25,000 s mm^−2^. This suggests the QDI technique is capable of observing a transition to an apparent power law but would be unable to define a final transition to an intraneurite power law as b→∞. Consequently, the QDI technique is sensitive to tissue heterogeneity as well as effects of semipermeable cell membranes in GM prior to transition to an intraneurite power law.

In WM, our negative power law exponents were higher than previously reported α≈0.5 [3, 12, 13] with the exception of the ex vivo data. Our in vivo results are consistent with higher exponents reported in WM for human participants analysed by QDI [33, 34, 35, 45]. Furthermore, they are consistent with negative power laws found in WM for water signal in diffusion MRS experiments estimated using the time‐fractional subdiffusion model (α=0.67) [78] than for total NAA, a metabolite exclusively found within neurons, for which the intraneurite stick model (α=0.5) provides excellent fits to brain tissue signal [79, 80]. Negative power law exponents estimated by QDI in our experiments were lower in data that were acquired with shorter δ (Dataset 1 δ/∆=12/23 ms, Dataset 2 δ/Δ=8/49 ms and Dataset 3 δ/∆=3.5/17.5 ms) potentially suggesting an effect of cell permeability on apparent α exponents estimated by QDI [24]. They were also higher in dMRI data acquired with longer ∆, further suggesting the sensitivity of QDI to cell permeability [24]. Theoretical modelling of water exchange between intracellular and intramyelin compartments suggests there are subsecond exchange times that can affect measured diffusion parameters, with greater permeability effects on ADC for longer effective diffusion times [81] and at higher b‐values [82, 83]. Cell permeability effects will break the intraneurite power law and increase observed negative power law exponents [13], an effect that has been demonstrated in simulation for presence in the presence of permeable sticks [84] and used to infer pathological mechanisms that include increased WM cell permeability in damaged axons due to multiple sclerosis [84]. An ex vivo mouse study of amyotrophic lateral sclerosis also reported α measured by QDI above 0.5 in affected mice [46] for dMRI acquired perpendicular to mouse spinal cord up to a maximum b‐value of 858,022 s mm^−2^ (δ/∆=11/400 ms). If a non‐negligible exchange between intracellular (which includes axons, oligodendrocytes, astrocytes, microglia and oligodendrocyte precursor cells [85], for which astrocytic processes cover approximately 48% of the voxel, a similar volume to myelin [85]) and extracellular spaces occur in healthy or pathological tissue during experimental diffusion times, the diffusion environment will not obey the intraneurite model until extremely high b‐values that are outside the experimental ranges analysed in our study. This suggests that QDI provides an apparent α that is sensitive to tissue heterogeneity and cell permeability in WM.

Our finding of time‐dependent QDI parameters in fixed rat brain tissue that tend towards Gaussian tortuosity limits as diffusion time increases indicates that QDI is sensitive to tissue heterogeneity and cell permeability. These results are comparable to findings of time‐dependent diffusion and kurtosis for in vivo GM [23, 26] and WM [28, 30, 32] and ex vivo tissue [24, 29, 31] and are further supported by observation of waiting time exponents increasing with diffusion time in application of the general CTRW model to the same ex vivo data as analysed in our study (Ingo et al. [49], for analysis of δ=3.5 ms, ∆=17.5 and 50 ms). Our results suggest that QDI provides tissue specific time dependence of $D_{1,2}$ and α such that tortuosity limits are approached faster in GM than WM, with the slowest changes occurring within the more restricted diffusion environment of the CC where an intra‐neurite power law was found at ∆=17.5 ms (median α=0.528) that slowly increased to α = 0.692 at ∆=200 ms. Our results do not indicate how high acquired b‐value ranges would have to be to observe transition to an intraneurite power law at long diffusion times up to 200 ms, but they suggest that QDI offers an alternative representation of time‐dependent diffusion to DKI from which tissue specific time dependence may be elucidated. Future studies will investigate the specificity of these time‐dependent effects in healthy and diseased tissue.

QDI parameters converge towards stable apparent values when maximum b‐values are approached in Dataset 1, indicating it provides an accurate representation of dMRI signal attenuation up to $b_{\max}=15,000$ s mm^−2^. Furthermore, α tends towards a Gaussian limit (α=1) as $b_{\max}\to 0$. Signal IPs have been previously shown to exist in GM signal [24] and can be identified using QDI. Although our signal IPs are a novel method for identifying tissue contrast between healthy tissue, they are a feature of the QDI functional form that is dependent on $D_{1,2}$ and α. As QDI provides a representation of dMRI signal, we caution against using this technique to make signal IP predictions outside experimentally acquired b‐value ranges [86]. This is particularly apparent in WM structures where low α leads to QDI IPs that are both shallow and located at b‐values higher than $b_{\max}$. Nevertheless, the QDI technique fits data exceptionally well over the analysed b‐value ranges suggesting that QDI can be used to identify a lower bound for the maximum b‐value that must be achieved at acquisition for accurate estimation of apparent signal power law behaviour. For directionally averaged signal in healthy tissue (with acquisition parameters δ/Δ = 12/23 ms, 0≤b≤15,000 s mm^−2^), this corresponded to $b_{\max}\geq 8000$ s mm^−2^; if lower maximum *b‐*values are acquired, such as on clinical MR scanners [33, 35], a systematic inaccuracy will be observed on α values for $b_{\max}\geq 3000$ s mm^−2^ that decreases with higher $b_{\max}$. We have shown here for dataset 1 that accurate QDI parameter estimation is possible for directionally averaged dMRI over 4 b‐values ($b=\left\{0,1200,4000,15000\right\}$ s mm^−2^) within a clinically feasible acquisition time of 6 min and 16 s. Future studies will optimise QDI acquisition protocols for a minimum of three b‐values [33] or from four b‐values for greater accuracy and precision of parameter estimation [35] to provide clinically feasible acquisition protocols based on data cohorts.

We have shown that QDI is a parsimonious representation of the diffusion decay curve and introduced properties of the signal IP, which is as an important feature of dMRI signal attenuation. By demonstrating that QDI provides an excellent fit to dMRI data of brain tissue over a wide range of diffusion times and b‐values from low to ultra‐high we have shown that it is sensitive to heterogeneous microstructure and semipermeable membranes. Furthermore, QDI provides time‐dependent parameters that may be specific to healthy and pathologically damaged tissue microstructure. QDI allows assessment of dMRI signal attenuation before its ultimate transition to an intraneurite power law. In this regime, the apparent negative power law exponent provided by QDI offers a more general approach to measurement of signal power laws than the intraneurite model alone and provides a continuous functional description of signal for b‐value ranges, diffusion gradient durations and diffusion times currently accessible in clinical research. This may enable rational development of clinically optimised acquisitions for tissue microstructural models as well as biomarkers of brain development, ageing and disease. As the technique provides stable, high SNR images that represent non‐Gaussian diffusion signal the technique will be sensitive to changes in the microstructural environment and have applications in disease diagnosis and monitoring disease progression.

## Conflicts of Interest

The QDI technique is covered by patent application GB1909982.9 published as WO 2021/005363 on 14 January 2021 (inventors: Dr T.R. Barrick, Prof F.A. Howe, Dr M.G. Hall, Dr C. Ingo and Prof R.L. Magin).

## Supporting information


**Data S1.** Supporting Information.

## Data Availability

The data that support the findings of this study will be available at City St George's, University of London, research repository (https://sgul.figshare.com/). The data were derived from the following resources available in the public domain (https://doi.org/10.17035/d.2022.0215863820 and https://doi.org/10.6084/m9.figshare.c.5315474). The ex vivo rat dataset is available on request.
