# Supplementary material for: Quasi‐Diffusion Imaging: Application to Ultra‐High b‐Value and Time‐Dependent Diffusion Images of Brain Tissue
Source: NMR Biomed. 2025 Feb 28;38(4):e70011. doi: 10.1002/nbm.70011 (PMC11868825; doi:10.1002/nbm.70011)
Supplement: Supplementary file 1 — Data S1. Supporting Information. [file NBM-38-e70011-s001.docx]

**Supplementary Material**

**Identification of the inflection point of the quasi-diffusion characteristic equation**

A functional form is derived that can be used to identify the $b$-value of the IP in the logarithm of the quasi-diffusion signal attenuation. All differentiation of the logarithm of the quasi-diffusion characteristic equation is performed with respect to $\ln\left( b \right)$. To derive the first and second derivatives of,

$$y=\ln\left( E_{\alpha,1}\left( -\lambda b^{\alpha} \right) \right), [A1]$$

with respect to $\ln\left( b \right)$ we initially substitute, $t=\ln\left( b \right)$ into Eq.A1 to give,

$$y=\ln\left( E_{\alpha,1}\left( -\lambda e^{t\alpha} \right) \right). [A2]$$

The chain rule for three nested functions is used to obtain the first derivative of Eq.A2 with respect to $t$ and states that,

$$\frac{dy}{dt}=\frac{dy}{du} \frac{du}{dv} \frac{dv}{dt} , [A3]$$

where in this case,

$$y=\ln\left( u \right), [A4]$$

$$u=E_{\alpha,1}\left( v \right), [A5]$$

$$v=-\lambda e^{t\alpha}. [A6]$$

The derivative of $y$ with respect to $u$ is,

$$\frac{dy}{du}=\frac{1}{u} , [A7]$$

with the derivative of $u$ with respect to $v$ given by Eq.9 as,

$$\frac{du}{dv}=\frac{E_{\alpha,0}\left( v \right)}{\alpha v}, [A8]$$

and the derivative of $v$ with respect to $t$ given by,

$$\frac{dv}{dt}=-\alpha\lambda e^{t\alpha}=\alpha v. [A9]$$

Substitution of Eqs. A7, A8 and A9 into Eq.A3 gives,

$$\frac{dy}{dt}=\frac{E_{\alpha,0}\left( -\lambda e^{t\alpha} \right)}{E_{\alpha,1}\left( -\lambda e^{t\alpha} \right)} . [A10]$$

and after substitution for $t$ the first derivative of Eq.A1 with respect to$\ln\left( b \right)$ is obtained,

$$\frac{dy}{d\left( \ln\left( b \right) \right)}=\frac{E_{\alpha,0}\left( -\lambda b^{\alpha} \right)}{E_{\alpha,1}\left( -\lambda b^{\alpha} \right)} . [A11]$$

The chain rule for two nested functions and the quotient rule are used to obtain the second derivative of Eq.A1 with respect to $t.$ Initially the chain rule is applied separately to the numerator and denominator of Eq.A10. For the numerator of Eq.A10 we have,

$$f=E_{\alpha,0}\left( -\lambda e^{t\alpha} \right), [A12]$$

with the nested functions given by,

$$f=E_{\alpha,0}\left( v \right), [A13]$$

$$v=-\lambda e^{t\alpha}. [A14]$$

The derivative of Eq.A13 with respect to $v$ is performed using Eq.A9,

$$\frac{df}{dv}=\frac{E_{\alpha,-1}\left( v \right)+E_{\alpha,0}\left( v \right)}{\alpha v}, [A15]$$

and the derivative of $v$ with respect to $t$ is given by Eq.A9. The chain rule then gives,

$$\frac{df}{dt}=\frac{df}{dv} \frac{dv}{dt}=E_{\alpha,-1}\left( -\lambda e^{t\alpha} \right)+E_{\alpha,0}\left( -\lambda e^{t\alpha} \right). [A16]$$

A similar method is used to obtain the derivative of the denominator of Eq.A10 such that,

$$g=E_{\alpha,1}\left( -\lambda e^{t\alpha} \right), [A17]$$

and,

$$\frac{dg}{dt}=E_{\alpha,0}\left( -\lambda e^{t\alpha} \right). [A18]$$

The quotient rule is used to obtain the second derivative of Eq.A1 with respect to $t$. The quotient rule states that for a function $h\left( t \right)=f(t)/g(t)$ where $f(t)$ and $g(t)$ are differentiable and $g(t)\neq0$ then,

$$h^{'}\left( t \right)=\frac{f^{'}\left( t \right)g\left( t \right)-f\left( t \right)g^{'}\left( t \right)}{\left( g\left( t \right) \right)^{2}}. [A19]$$

In our case, $h\left( t \right)$ is given by Eq.A10. By substituting Eqs. A12, A16, A17 and A18 into Eq.A19, the second derivative of Eq.A1 with respect to $t$ is obtained,

$$\frac{d^{2}y}{{dt}^{2}}=\frac{\left( E_{\alpha,-1}\left( -\lambda e^{t\alpha} \right)+E_{\alpha,0}\left( -\lambda e^{t\alpha} \right) \right)E_{\alpha,1}\left( -\lambda e^{t\alpha} \right)-\left( E_{\alpha,0}\left( -\lambda e^{t\alpha} \right) \right)^{2}}{\left( E_{\alpha,1}\left( -\lambda e^{t\alpha} \right) \right)^{2}}, [A20]$$

and after substitution for $t$ we have,

$$\frac{d^{2}y}{{d\left( \ln\left( b \right) \right)}^{2}}=\frac{\left( E_{\alpha,-1}\left( -\lambda b^{\alpha} \right)+E_{\alpha,0}\left( -\lambda b^{\alpha} \right) \right)E_{\alpha,1}\left( -\lambda b^{\alpha} \right)-\left( E_{\alpha,0}\left( -\lambda b^{\alpha} \right) \right)^{2}}{\left( E_{\alpha,1}\left( -\lambda b^{\alpha} \right) \right)^{2}}. [A21]$$

The inflection point occurs when,

$$\frac{d^{2}y}{{d\left( \ln\left( b \right) \right)}^{2}}=0, [A22]$$

and satisfies the following equation,

$$\left( E_{\alpha,-1}\left( -\lambda b^{\alpha} \right)+E_{\alpha,0}\left( -\lambda b^{\alpha} \right) \right)E_{\alpha,1}\left( -\lambda b^{\alpha} \right)-\left( E_{\alpha,0}\left( -\lambda b^{\alpha} \right) \right)^{2}=0. [A23]$$
